# Benchmark classification dataset for laser-induced breakdown spectroscopy

**DOI:** 10.1038/s41597-020-0396-8

**Published:** 2020-02-13

**Authors:** Erik Képeš, Jakub Vrábel, Sára Střítežská, Pavel Pořízka, Jozef Kaiser

**Affiliations:** Central European Institute of Technology, Brno University of Technology, Purkyňova 123, 612 00 Brno, Czech Republic

**Keywords:** Optical spectroscopy, Optical techniques, Analytical chemistry

## Abstract

In this work, we present an extensive dataset of laser-induced breakdown spectroscopy (LIBS) spectra for the pre-training and evaluation of LIBS classification models. LIBS is a well-established spectroscopic method for *in-situ* and industrial applications, where LIBS is primarily applied for clustering and classification tasks. As such, our dataset is aimed at helping with the development and testing of classification and clustering methodologies. Moreover, the dataset could be used to pre-train classification models for applications where the amount of available data is limited. The dataset consists of LIBS spectra of 138 soil samples belonging to 12 distinct classes. The spectra were acquired with a state-of-the-art LIBS system. Lastly, the composition of each sample is also provided, including estimated uncertainties.

## Abstract (summary)

**Table:** 

Measurement(s)	emission spectrum
Technology Type(s)	laser-induced breakdown spectroscopy
Factor Type(s)	class
Sample Characteristic - Environment	soil

Machine-accessible metadata file describing the reported data: 10.6084/m9.figshare.11799207

## Background & Summary

Laser-induced breakdown spectroscopy (LIBS) is an emission spectroscopic method that uses a high-powered laser pulse to ignite a microplasma. This is achieved by focusing laser pulses with lengths in the fs–ns range into spots with diameters of tens of µm. Consequently, a sufficiently high energy fluence is reached to ionize the target material. Subsequently, emission of the ignited plasma is collected, dispersed by a spectrometer, and recorded. Assuming a stoichiometric ablation, the dispersed light intensities can be related to the composition of the target material^1,2^.

Owing to these relatively simple principles, LIBS instrumentations are generally robust. Consequently, LIBS is often preferred in industrial settings that are unfavourable for most common spectroscopic methods, such as charged-particle-based techniques and variations of mass spectrometry. As such, LIBS has been widely adapted in geology^3,4^, steel industry^5^, and forensics^6^. Nevertheless, recently, LIBS has been gaining a foothold in various biological applications, e.g., mapping of biological samples^7,8^.

Generally, most successful applications of LIBS are clustering and classification^9,10^. Meanwhile, the current limitations of LIBS inhibit LIBS from being reliably applied for quantification. Consequently, there is a relatively wide range of literature reporting on the classification of various materials using LIBS. As such, the classification approaches also vary significantly, including the spectral pre-processing, feature engineering, the classification model itself^11^. Hence, the systematic comparison of the various approaches is not possible. Moreover, a common approach to classification is the randomized division of the complete dataset into training, validation, and testing subsets. Hence, this approach relies on the testing dataset comprising emission spectra that were collected during the same measurement as the training spectra. However, in practical applications, fresh data is constantly being evaluated by the existing model, i.e., the testing dataset is constantly evolving.

**Fig. 1:**
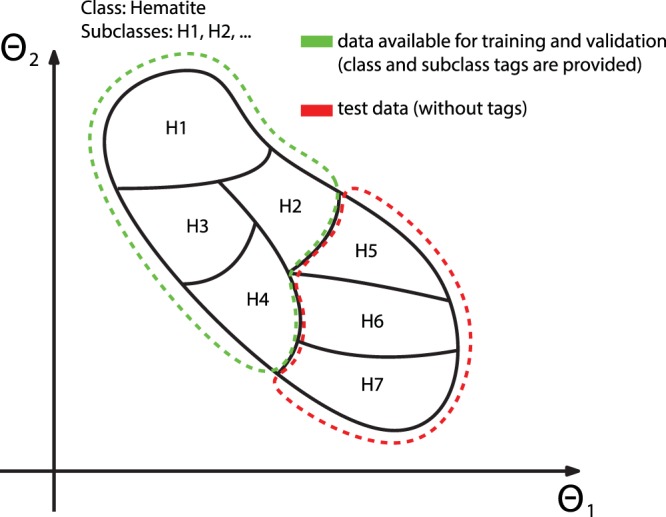
Schematic representation of the classification task: samples belonging to the geological class of hematite in an arbitrary two-dimensional feature space.

Consequently–inspired by the recent breakthroughs in image recognition tasks partially made possible by datasets such as MNIST handwritten digit dataset^12^–we propose a similar dataset for LIBS. The dataset is constructed from geological samples, where several distinct samples belong to the same class. Thus, we propose a dataset where the training and testing data is sampled from distinct materials. As such, classifiers that perform well on the proposed dataset must be able to generalize rather than simply learn the distribution of the data. The classification problem is shown schematically in Fig. 1: Various ore samples belong to the same geological class, e.g., the class hematite is represented by six samples. Four of these samples are provided in the training dataset, while the remaining two are included in the test dataset. Considering the interclass variability, this classification task is more challenging than the generally reported cases since the model is expected to generalize from the samples in the green region in order to accurately classify the samples in the red region.

The dataset is expected to provide not only a benchmark for LIBS classification models but also the means of testing classification models for robustness or to identify overtraining. Moreover, the dataset could prove useful for implementing transfer learning for applications where only relatively small datasets are available. Lastly, the dataset could be used to develop feature-engineering and dimensionality-reduction methodologies.

## Methods

### Sample preparation

The samples comprised certified reference materials (soils) purchased from Ore Research & Exploration Pty Ltd (Melbourne, Australia) and dental gypsum (Spofadental, Czechia) in a weight ratio of 1:1, i.e., each sample consisted of 50 wt.% certified reference soil powder (see below) and 50 wt.% gypsum powder. The samples were prepared by mixing 400 mg of dry soil powder and 400 mg of dry gypsum powder. The weight of the constituents was measured with a TP-303 laboratory scale (Denver Instruments, Germany) with an instrumental uncertainty of ±1 mg. In total, 46 soil samples were used (table included in the data repository). Each of the 46 soil powders belongs to one of 12 ore types. The latter classification was provided by the vendor and can be found in the *OREAS* tab of the *support_tables.xlsx* excel file accessible in the data repository. For simplicity, in the upcoming description of the sample preparation, the gypsum portion of the samples is considered to be constant (within the measurement uncertainty) and is excluded.

To adjust the classification difficulty of the dataset in a controlled manner, the soil powders were mixed to obtain a certain degree of intraclass similarity: Each of the 46 soil samples (base sample) was mixed with two soil powders (additive samples) from a different ore class. Consequently, 138 samples were obtained in total. During the mixing, ¼ of the base sample’s mass was replaced with one of the additives. Subsequently, 0.5 ml water was added followed by another mixing. Lastly, the wet mixture was poured into a small plastic container, where it formed a flat surface.

Although the dataset is not meant for quantitative analysis, the composition of the samples is provided in the data repository including estimated uncertainties. The uncertainties are provided without considering the influence of the added water.

### Measurements

The samples were measured in a state-of-the-art LIBS interaction chamber that enables the precise control of the measurement parameters, including the atmosphere. As such, the highest standards of LIBS measurements were maintained. The samples were mapped with a 100 µm step size (distance between shots) at a 20 Hz ablation repetition rate with a pulse energy of 15 mJ at the ablation wavelength of 532 nm (Nd:YAG, 10 ns pulse length, CFR400, Quantel, France). The ablation crater diameter measured under an optical microscope was 60 µm. The optical emission of the laser-induced plasma was collected using a single lens and guided to the entrance slit of an echelle spectrograph (EMU 65, Catalina Scientific, US; resolving power *R *= 6000) by an optical fibre. The light resolved by the spectrometer (the echellogram) was recorded with an EMCCD camera (Falcon Blue, Raptor Photonic, IR) and translated into spectra using the control software proprietary to the spectrometer (KestrelSpec™ Imaging Spectroscopy, Catalina Scientific, US). The camera recorded the incoming light with a delay of 0.3 µs after the ablation laser pulse (commonly referred to as the gate delay) and for the duration of 50 µs (commonly referred to as the gate width). These timing values have been chosen following an optimization procedure based on the signal-to-baseline ratio, where the height of an arbitrary emission line of a non-matrix element has been considered as the signal. The measurements were carried out in air. Moreover, during the measurements, the sample surface was continuously purged by air with a volumetric flow rate of 10 l/min.

## Data Records

The dataset (available on Figshare^13^) consists of two hdf5 files, a .csv file and a .xlsx table. The first hdf5 file (*train.h5*) contains the training dataset which is advised to be further divided into a training and validation dataset. The training dataset includes class labels. The second hdf5 (*test.h5*) file contains the testing data without class labels. The rows of each dataset correspond to a single emission spectrum obtained by laser-induced breakdown spectroscopy. Consequently, the columns correspond to distinct wavelength values and the elements of the dataset are intensity values in arbitrary units (a.u.), which is a common representation of emission intensity in the LIBS community. The training dataset contains 500 spectra for each sample. Nevertheless, the users are welcome to load in only a subset of the dataset (which is straightforward with the supported code). Meanwhile, the testing dataset contains a varying number of spectra for each sample.

The class labels of the testing dataset are provided in the form of a .csv file titled *test_labels .csv*. Lastly, the composition of the samples is given in an .xlsx file (*support_tables.xlsx*). The excel file contains 4 spreadsheets: *OREAS* lists the composition of the certified soil powders as provided by the vendor; *MIXED_composition* lists the estimated composition of the mixed samples (excluding the gypsum fraction); *MIXED_uncertainty* lists the estimated uncertainties of the mixed compositions; and *MIXED_combined* lists the mixed compositions and uncertainties in a more compact form. The aim of providing the composition and the uncertainties in separate tables is to ease their import for data processing. Consequently, the table combining both the composition and uncertainties facilitates the presentation of the compositions.

## Technical Validation

The composition of the samples is provided with relevant uncertainties in the data repository. The soil samples are certified standard materials. Hence, their composition was determined by the vendor. The uncertainty of the constituents’ weight fraction ranges from 4 to 10%. However, for a more modest uncertainty estimation, a constant uncertainty of 10% was considered, e.g., the uncertainty of an element present in the soil with a weight fraction of 10 wt.% was ±1 wt.%. The final combined uncertainty was determined from the non-linear uncertainty propagation as:

$${\Delta }_{f}=\sqrt{\mathop{\sum }\limits_{i=1}^{N}{\left(\frac{\partial {f}_{e}({x}_{1},\ldots ,{x}_{N})}{\partial {x}_{i}}\Delta \left({x}_{i}\right)\right)}^{2}}$$

where ${\Delta }_{f}$ is the combined uncertainty, $\Delta \left({x}_{i}\right)$ is the uncertainty of *x*_*i*_, ${f}_{e}({x}_{1},\ldots ,{x}_{N})$ is the function describing the weight fraction of an analyte *e* in the final sample; namely:

$${f}_{e}\left({x}_{1},\,\ldots ,{x}_{N}\right)={W}_{e}=\frac{{M}_{s,1}\cdot {w}_{e,1}+{M}_{s,2}\cdot {w}_{e,2}}{{M}_{s,1}+{M}_{s,2}+{M}_{G}},$$

where *W*_*e*_ is the weight fraction of analyte *e* in the sample; ${M}_{s,k}$ and ${w}_{e,k}$ are the weight of soil sample *k* and the analyte’s weight fraction in soil *k*, respectively; and *M*_*G*_ is the weight of the added gypsum powder. For samples mixed from a single soil standard, ${M}_{s,2}=0$. Consequently, the uncertainty of analyte *e* in the final sample is given as:

$$\Delta \left({W}_{e}\right)=\sqrt{\begin{array}{c}{\left(\frac{{w}_{e,1}\cdot ({M}_{s,1}+{M}_{s,2}+{M}_{G})-{M}_{s,1}\cdot {w}_{e,1}}{{({M}_{s,1}+{M}_{s,2}+{M}_{G})}^{2}}\cdot \Delta ({M}_{s,1})\right)}^{2}+\\ +{\left(\frac{{w}_{e,2}\cdot ({M}_{s,1}+{M}_{s,2}+{M}_{G})-{M}_{s,2}\cdot {w}_{e,2}}{{({M}_{s,1}+{M}_{s,2}+{M}_{G})}^{2}}\cdot \Delta ({M}_{s,2})\right)}^{2}+\\ +{\left(\frac{{M}_{s,1}}{{M}_{s,1}+{M}_{s,2}+{M}_{G}}\cdot \Delta \left({w}_{e,1}\right)\right)}^{2}+\\ +{\left(\frac{{M}_{s,2}}{{M}_{s,1}+{M}_{s,2}+{M}_{G}}\cdot \Delta \left({w}_{e,2}\right)\right)}^{2}+\\ +{\left(\frac{{M}_{s,1}\cdot {w}_{e,1}+{M}_{s,2}\cdot {w}_{e,2}}{{({M}_{s,1}+{M}_{s,2}+{M}_{G})}^{2}}\cdot \Delta ({M}_{G})\right)}^{2}\end{array}}$$

where $\Delta \left(X\right)$ is the uncertainty of the quantity *X*. Lastly, the uncertainty of the weight fraction of element *e* in the soil sample *k* is determined as $\Delta \left({w}_{e,1}\right)=0.1\cdot {w}_{e,k}$.

The dataset was classified as part of a competition held at the EMSLIBS19 conference (http://libs.ceitec.cz/libs-contest/). The highest accuracy achieved was approximately 90%. Two additional approaches reached classification accuracies over 80%. Details of the applied methodologies will be specified elsewhere. Nevertheless, the classification of the dataset has been proven to be adequately challenging to serve as a benchmark dataset.

## Data Availability

Custom code for loading in the training and testing datasets is available in the data repository for Python, R, and MATLAB. The Python code was tested in Python 3.6 and requires the following libraries: “os”, “h5py”, and “numpy”. The R code was tested in R 3.5.2 and requires the following libraries: “rhdf5”. Lastly, the MATLAB code was tested in MATLAB 2016. The codes are intended to load in the data from the hdf5 files in a user-friendly manner.
